# The Impact of a History of Different Other Cancers on the Long-Term Outcomes of Patients with Intrahepatic Cholangiocarcinoma: A Population-Based Analysis

**DOI:** 10.1155/2022/3970884

**Published:** 2022-02-25

**Authors:** Jiawei Chai, Junjie Kong, Kunbing Zhu

**Affiliations:** ^1^Department of Breast and Thyroid Surgery, Shandong Provincial Maternal and Child Health Care Hospital, Jinan, Shandong Province, 250014, China; ^2^Department of Liver Transplantation and Hepatobiliary Surgery, Shandong Provincial Hospital, Cheeloo College of Medicine, Shandong University, Jinan, Shandong Province, 250021, China; ^3^Department of Liver Transplantation and Hepatobiliary Surgery, Shandong Provincial Hospital Affiliated to Shandong First Medical University, Jinan, Shandong 250021, China

## Abstract

**Background:**

The characteristics and outcomes of patients with intrahepatic cholangiocarcinoma (ICC) with prior malignancy are poorly clarified. This study is aimed at exploring the impact of prior malignancy on the long-term outcomes of ICC patients.

**Methods:**

Using the Surveillance, Epidemiology, and End Results (SEER) program, ICC patients diagnosed between 2004 and 2018 were identified. Kaplan-Meier curves and Cox analysis were used to evaluate the impact of prior malignancy on the prognosis of ICC patients.

**Results:**

A total of 9667 ICC patients were identified; among them, 782 (8.09%) had a history of prior cancer. Prostate, breast, colorectal, bladder, and liver/gallbladder/other biliary cancers were the most common types of prior cancer. Patients with prior cancer had better tumor-related profiles than those without prior cancer, namely, the former patients showed a lower proportion of positive AFP levels and vascular invasion, a lower AJCC stage, a smaller tumor size, and a lower stage of tumor grade. The median survival times after the diagnosis of ICC were 10 and 11.5 months for patients with and without prior cancer, respectively. Multivariate regression analysis suggested that prior cancer did not contribute to inferior overall survival (OS, HR 0.870, 95% CI 0.797-0.950, and *p* = 0.002) or cancer-specific survival (CSS, HR 0.820, 95% CI 0.741-0.906, and *p* < 0.001).

**Conclusions:**

A history of prior cancer does not lead to worse OS or CSS for ICC patients. The exclusion of patients with prior cancer from clinical trials should be reconsidered.

## 1. Introduction

Intrahepatic cholangiocarcinoma (ICC), arising from the epithelial cells of the intrahepatic bile ducts, is the second most frequent primary liver malignancy [1]. During the last few decades, the incidence of ICC has been on the rise worldwide; in the United States, the incidence of ICC has increased from 0.44 to 1.18 cases per 100,000 person-years over the past 40 years [2]. However, since the majority of ICC patients are usually diagnosed at an advanced stage, limited patients are eligible for curative treatment, and the prognosis of ICC is poor. The 5-year OS for ICC ranges from 21% to 35% [3]. While various efforts have been made to advance the diagnosis and treatment of primary ICC, little is known about the clinical characteristics, prognostic factors, and clinical significance of ICC as a subsequent cancer with other types of prior malignancy.

Due to early diagnosis and advancements in therapy, the prognosis of malignancies has been greatly improved. The number of cancer survivors is growing and is estimated to be 26.1 million by 2040 in the United States [4]. During a relatively long-term survival period, cancer survivors are at risk of developing subsequent malignancies [5, 6]. In a recent population-based study, the incidence of hepatocellular carcinoma (HCC) and ICC in patients with prior cancer was reported to be 5.0% and 17.0% for patients diagnosed at an age of <65 and ≥65 years old, respectively [7]. Consequently, understanding the characteristics and prognosis and exploring appropriate surveillance and therapy strategies for these patients are important. However, because prior malignancies could influence the implementation and efficiency of treatment, patients with prior malignancy are commonly excluded from clinical trials, and little is known about the characteristics of these patients. In addition, whether prior cancer influences the long-term survival of ICC patients is poorly understood.

In this study, using data obtained from the Surveillance, Epidemiology, and End Results (SEER) program, we comprehensively analyzed the demographic and clinical characteristics of ICC patients with a history of prior malignancies. Furthermore, the clinical outcomes of ICC patients with prior malignancy were also analyzed. The findings in this study might provide potential implications for the management and surveillance of ICC patients with prior malignancy.

## 2. Materials and Methods

### 2.1. Data Source and Population Selection

This study was approved by the Ethics Committee of Shandong Provincial Maternal and Child Health Care Hospital and was performed in accordance with the Declaration of Helsinki (as revised in 2013). The SEER Research Plus Data, 18 Registries, Nov 2020 Sub (2000-2018), was used to obtain clinical data; the SEER Research Data, 18 Registries (excl AK), Nov 2020 Sub (2000-2018), was used to obtain details of multiple cancers; the SEER∗Stat software (version 8.3.9) was used for data exploration. Using the cite code C22.1 (intrahepatic bile duct) and a histological diagnosis of cholangiocarcinoma (International Classification of Disease for Oncology, 3^rd^ Edition [ICD-O-3], 8160 (cholangiocarcinoma) or 8140 (adenocarcinoma)) or the cite code C22.0 (liver) and histological code 8160, patients diagnosed with ICC between 2004 and 2018 were identified. The exclusion criteria were as follows: (1) patients diagnosed on death autopsy or death certificate only, (2) patients with an unknown survival time or follow-up less than 1 month, and (3) patients diagnosed with ICC who were aged <18 or >90 years old. In addition, to avoid the possibility of synchronous primary cancer or metastasis, a latency of 6 months was required after the diagnosis of initial malignancies. Subsequently, eligible patients were divided into two groups: the ICC with prior malignancy (ICC-PM) group and the ICC only (OICC) group.

### 2.2. Outcomes and Variables

The follow-up time lasted to December 31, 2018. Overall survival (OS) was defined as the time from the diagnosis of ICC to death of any cause. Cancer-specific survival (CSS) referred to the time from ICC diagnosis to death due to ICC. Demographic characteristics included age at diagnosis (<65 and ≥65), sex, race (white, black, and others), and marital status (single, married, and unknown). Tumor-related factors included alpha-fetoprotein (AFP) (negative, positive, and unknown), size (0-5 cm, 5-8 cm, >8 cm, and unknown), vascular invasion (no, yes, and unknown), grade (I/II, III/IV, and unknown), and AJCC stage (I, II, III, IV, and unknown). Treatment-related variables included treatment (none, local tumor destruction, surgery, and unknown), radiation (no, yes, and unknown), and chemotherapy (no, yes, and unknown).

### 2.3. Statistical Analysis

Continuous covariates were expressed as medians and interquartile ranges (Q1-Q3), and categorical variables were expressed as numbers (*n*) and proportions (%). To compare the difference between the ICC-PM and OICC groups, the Mann-Whitney *U*-test was used for continuous variables, and the chi-square test and Fisher's exact test were used for categorical covariates. Kaplan-Meier analysis and the log-rank test were used to compare the OS and CSS between the ICC-PM and OICC groups. Cox regression analysis was used to explore independent risk factors for the prognosis of ICC. Covariates with *p* < 0.05 in the univariate analysis were considered potential prognostic factors and were included in the multivariate analysis. The hazard ratios (HRs) and 95% confidence intervals (95% CIs) were recorded. To confirm the reliability of our findings, subgroup analysis was performed to evaluate the influence of a history of prior malignancy on the long-term survival of ICC stratified by AJCC stage, latency (<60 months and ≥60 months) and various types of prior cancer.

SPSS version 26.0 (SPSS Inc., Chicago, IL) and R software (version 3.5.2) were used in the statistical analysis. All statistical tests were two-sided, and *p* < 0.05 was considered to indicate statistical significance.

## 3. Results

### 3.1. Patients' Baseline Characteristics

A total of 9667 patients with ICC were identified from the SEER database based on the inclusion criteria; of these patients, 782 (8.09%) had a history of other types of prior cancer, including 142 (1.5%) patients who had a history of multiple malignancies. The demographic and clinical characteristics of the eligible patients are displayed in Table 1. Compared to those in the OICC group, more patients in the ICC-PM group were older, more were male, and more were white. Interestingly, we found that ICC patients with prior malignancy had better tumor-related profiles than those without prior cancer, namely, the former patients showed a lower proportion of positive AFP levels and vascular invasion, a lower AJCC stage, a smaller tumor size, and a lower stage of tumor grade. Furthermore, patients with prior cancer were more likely to receive tumor-related treatment than those without prior cancer.

**Figure 1:**
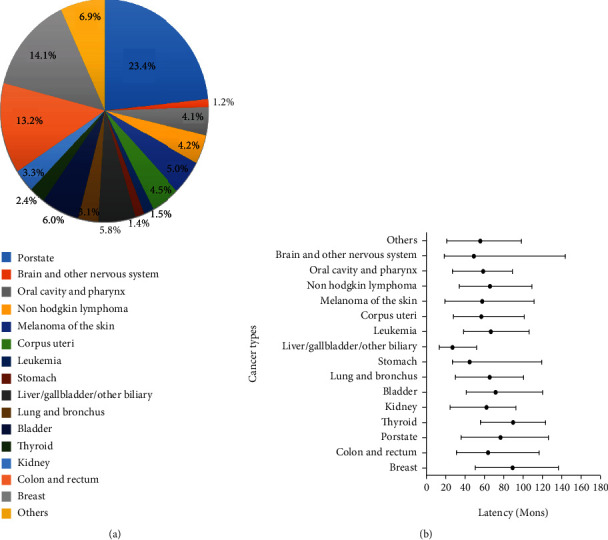
(a) Distribution of prior cancer among patients with ICC and (b) distribution of the median interval time from prior cancer diagnosis to the subsequent ICC diagnosis. ICC: intrahepatic cholangiocarcinoma.

The distribution of ICC with prior malignancy is shown in Figure 1(a). We found that the most common prior cancers were prostate (23.4%), breast (14.1%), colon and rectum (13.2%), bladder (6.0%), and liver/gallbladder/other biliary (5.8%) cancers. As shown in Figure 1(b), the median latency from the diagnosis of prior malignancy to the development of ICC was 66 months, ranging from liver/gallbladder/other biliary cancer (21 months) to breast cancer (89.5 months).

### 3.2. Comparison of OS and CSS in ICC Patients with and without Prior Cancer

The median survival times after the diagnosis of ICC were 10 and 11.5 months for the ICC-PM and OICC groups, respectively. The cause of death is shown in Figure S1. During the follow-up period, a total of 7608 (78.7%) patients died, and the proportions of patients who died of cancer in the OICC and ICC-PM groups were 90.6% and 93.3%, respectively. Patients in the OICC group were more likely to die from ICC than those in the ICC-PM group (90.56% vs. 75.36%).

**Figure 2:**
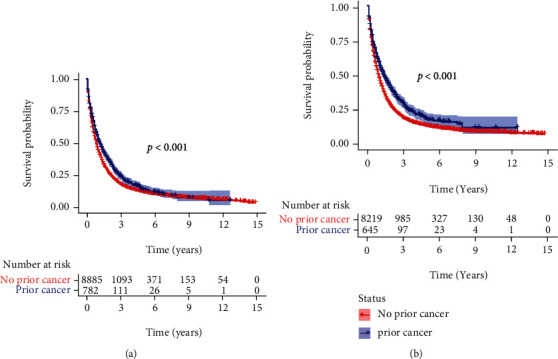
The OS and CSS of intrahepatic cholangiocarcinoma patients with and without prior cancer. (a) OS; (b) CSS. OS: overall survival; CSS: cancer-specific survival.

**Figure 3:**
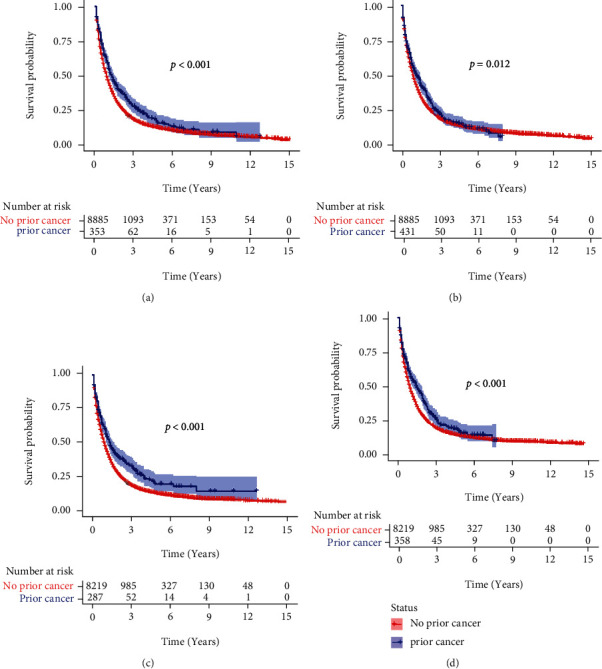
The OS and CSS of subgroups stratified by time latency. (a) Latency < 60 months and (b) ≥60 months for OS analysis; (c) latency < 60 months and (d) ≥60 months for CSS analysis. OS: overall survival; CSS: cancer-specific survival.

**Figure 4:**
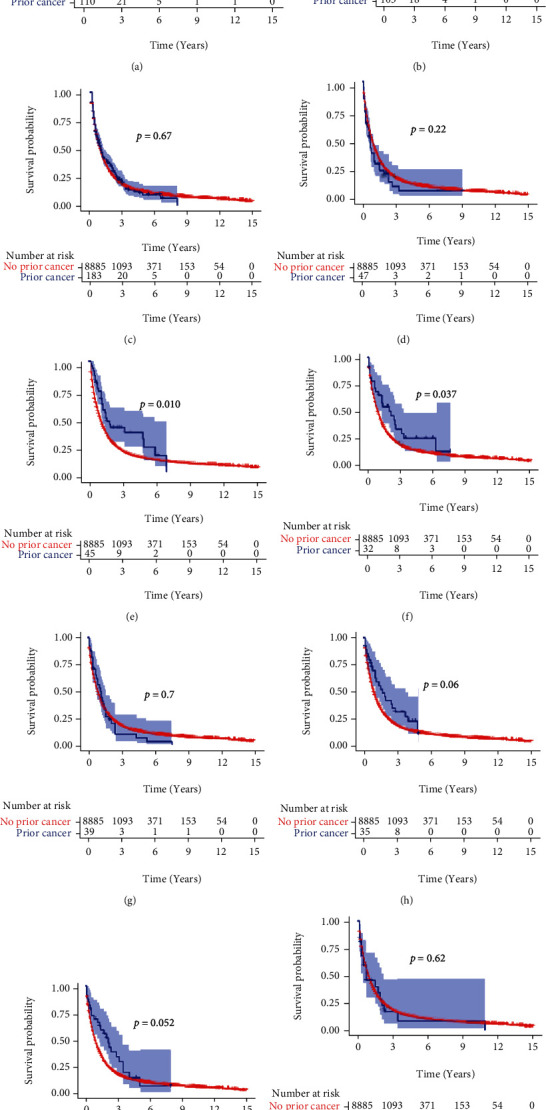
OS of subgroups stratified by prior cancer site. (a) Breast cancer, (b) colorectal cancer, (c) prostate cancer, (d) bladder cancer, (e) gallbladder/HCC/other biliary, (f) oral cavity and pharynx cancer, (g) melanoma, (h) uteri, (i) non-Hodgkin lymphoma, and (j) kidney. OS: overall survival; HCC: hepatocellular carcinoma.

**Figure 5:**
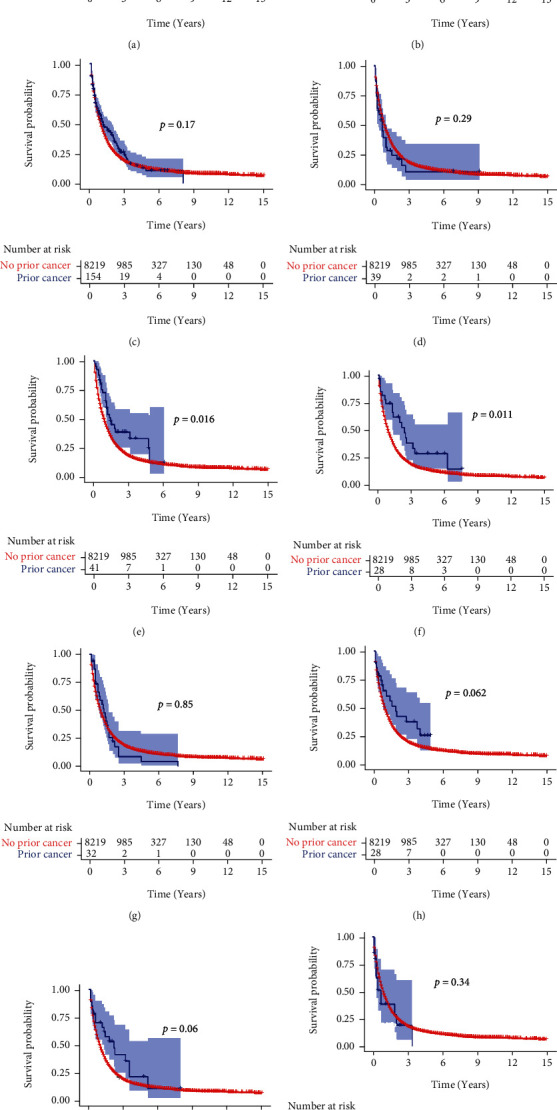
The CSS of subgroups stratified by prior cancer site. (a) Breast cancer, (b) colorectal cancer, (c) prostate cancer, (d) bladder cancer, (e) gallbladder/HCC/other biliary, (f) oral cavity and pharynx cancer, (g) melanoma, (h) uteri, (i) non-Hodgkin lymphoma, and (j) kidney. OS: overall survival; HCC: hepatocellular carcinoma.

The 1-, 3-, and 5-year OS and CSS for the OICC and ICC-PM groups are shown in Table S1. The Kaplan-Meier analysis showed that ICC patients with prior cancer had better OS and CSS than those without prior cancer (Figures 2(a) and 2(b), *p* < 0.001). In the subgroup analysis, when patients were stratified by time latency, ICC patients with prior cancer had better OS than those without prior cancer regardless of latency < 60 months (*p* < 0.001) or ≥60 months (*p* = 0.012) (Figures 3(a) and 3(b)). Similar results were also found in CSS (Figures 3(c) and 3(d)). When stratified by AJCC stage, only AJCC I stage ICC patients with prior cancer had better OS and CSS than those without prior cancer (*p* = 0.035 and *p* < 0.001 for OS and CSS, respectively) (Figure S2 and S3). When stratified by initial cancer site, compared to those without prior cancer, the survivors of breast, colon and rectum, liver/gallbladder/other biliary, and oral cavity and pharynx cancer had better OS (Figure 4) and CSS (Figure 5), while the survivors of other types of prior cancer had comparable OS and CSS between the two groups.

### 3.3. Identification of Independent Prognostic Factors for OS and CSS

To explore the independent prognostic factors for OS and CSS, a Cox proportional hazard model was used.  Table 2 shows the results of univariate and multivariate Cox regression analyses of the included ICC patients. For OS, we found that prior cancer, age at diagnosis, sex, marital status, AFP level, AJCC stage, tumor size, tumor grade, treatment, radiation, and chemotherapy were independent prognostic factors. Prior cancer was associated with prolonged OS (HR 0.870, 95% CI 0.797-0.950, and *p* = 0.002). Independent prognostic factors for CSS were the same as those for OS, and prior cancer was still found to be significantly associated with prolonged CSS (HR 0.820, 95% CI 0.741-0.906, and *p* < 0.001). Furthermore, as shown in Table 3, in the subgroup analysis stratified by different types of prior cancer, only prior breast (HR 0.748, 95% CI 0.574-0.975, and *p* = 0.032) and prostate (HR 0.786, 95% CI 0.649-0.953, and *p* = 0.014) cancer was found to be associated with prolonged CSS.

## 4. Discussion

In recent decades, the number of cancer survivors has been increasing rapidly [4]. Cancer survivors have special health needs, including treatment-related toxicities [8] and recurrence of disease [9]; moreover, the incidence of multiple primary malignancies has also been growing rapidly and is a threat to the health of cancer survivors [10, 11]. However, since the majority of previous trials excluded populations with prior cancer from cohort enrollment, few studies have focused on these patients [7, 12, 13]. Consequently, limited evidence is available to guide appropriate surveillance and therapy for these patients. To the best of our knowledge, no study has reported the characteristics and prognosis of ICC patients with a history of prior cancer, and there is a need for attention to be given to these patients.

In this study, using data from the SEER database, a total of 782 ICC patients with prior cancer were identified. The clinical characteristics of these patients were analyzed, and we found that compared to ICC patients without prior cancer, those with prior cancer did not have inferior OS or CCS. Subgroup analysis also gave similar results. These findings were similar to those in previous studies focused on other cancers [13–15]. Therefore, the following statement of the NCI Cancer Therapy Evaluation Program (CTEP) seems reasonable. Individuals who have received curative therapy for prior malignancy and did not have tumor recurrence for 5 years could be a participant in a cancer treatment trial for a subsequent cancer [16].

Several interesting findings were identified in this study. First, we found that among the ICC patients with prior cancer, the prostate, breast, and colorectum were the top three most common sites. Similar results were also found in studies focused on other cancers [12, 14, 17]. This could be explained by the following reasons: (1) all three types of cancer have a high incidence, and in a recent study, breast, colorectal, and prostate cancer were reported to be the first, third, and fourth most commonly diagnosed malignancies worldwide, respectively [18]; (2) the indolent characteristics of the three types of cancer and the advancement in therapeutic strategies contribute to a prolonged OS for patients [19–21], and the relatively long-term survival time results in an increasing chance of developing a second malignancy. However, lung cancer, which is the second most commonly diagnosed cancer [18], has a low incidence of second malignancy in cancer survivors because of the high fatality rate [22]. This might be explained by the relatively poor survival and prognosis of lung cancer [23].

Another interesting finding in this study was that compared to those without prior malignancy, ICC patients with prior cancer had significantly different clinical characteristics. First, we found that ICC patients with prior cancer were older (>65 years), and similar results were also found in studies focusing on other cancers [24, 25]. This phenomenon might be associated with changes in the immune system; it was reported that older age was associated with decreased immunity (“immunosenescence”), which could contribute to increased tumorigenesis. In addition, ICC patients with prior cancer were associated with better tumor-related factors. We thought the main reason for this was that the majority of these patients had regular surveillance of their health. It is well known that the insidious onset of ICC usually leads to a late diagnosis of disease, which causes patients to miss an opportunity to receive an appropriate treatment. The regular surveillance of disease could make the diagnosis of disease (especially cancer) more prompt. In HCC, Xu et al. found that patients who underwent regular postoperative surveillance had significantly better OS than those who did not [26]. Ladigan-Badura et al. also found that through regular upper gastrointestinal endoscopy surveillance, clinicians could observe gastric cancer earlier, which could contribute to a better oncological outcome [27]. Consequently, for those at high risk of developing ICC, regular and active surveillance might lead to an early diagnosis of disease, and patients might benefit from long-term survival.

Finally, we found that prior cancer did not contribute to a worse prognosis for cancer survivors. In the included patient cohort, ICC patients with prior cancer were found to have better OS and CSS than those with only ICC. The following reasons could explain this phenomenon: (1) ICC patients with prior cancer had better tumor-related factors, which might lead to a better long-term outcome; (2) ICC patients with prior cancer might be under reduced exposure to risk factors for developing cancer, such as alcohol and smoking; (3) these patients might have better compliance with surveillance and treatment. A main reason for the exclusion of patients with prior cancer from clinical trials was the assumption that previous malignancies could influence oncological outcomes [28]. However, recent studies have found comparable long-term outcomes between patients with and without prior cancer [14, 15, 29]. In addition, for patients with lung cancer with prior cancer, recent studies have challenged the rationality of excluding these patients and have started to reconsider the design of current clinical trials [12, 13]. Overly restrictive restrictions in patient selection might lead to a low rate of enrollment, and the number of participants could be insufficient; meanwhile, it could lead to a loss of generalizability of trial results and to a limitation of the ability to understand the benefit-risk profile of the therapy in patients who might receive the intervention [30]. Furthermore, since there is a difference in survival rates and tumor-related factors between patients with and without a history of prior different other cancers, it could lead to a study bias when including these patients in clinical trials. However, as discussed above, this is caused by the difference in factors such as surveillance, treatment, and exposure to risk factors for developing cancer, which could be avoided by rigorous patient selection criteria to some extent. Based on the increasing number of cancer survivors, combined with the findings in this and previous studies, when OS is the major study endpoint, the exclusion of patients with prior cancer should be reconsidered. Further studies focusing on this topic should be conducted.

There are several limitations in our study. First, due to the nature of the SEER database, several well-known cancer risk factors, such as smoking, alcohol consumption, and family history, and prognostic factors for ICC, including CA199 and CEA, were unavailable, which might lead to a bias of our results. However, using a large number of patients and appropriate statistical methods, we still preliminarily analyzed the characteristics of ICC patients with prior cancer and obtained the prognostic information of these patients. Second, apart from the sequence number, latency, and site of prior cancer, we could not obtain any clinical profiles of the prior cancers, and these factors might be associated with the prognosis of patients. Third, the SEER database only contained a population from the U.S., whether the findings of this study are applicable to patients in other countries, such as those with a relatively high incidence of ICC, is uncertain [31, 32], and there is still a need for an external patient cohort to further validate our findings.

## 5. Conclusions

In conclusion, the demographic and clinical characteristics of ICC patients with prior cancer were significantly different from those without prior cancer. Prior cancer did not contribute to a worse prognosis for cancer survivors. The exclusion of patients with prior cancer from clinical trials should be reconsidered. Further studies are needed to validate our findings.

## Figures and Tables

**Table 1:** Baseline characteristics of included ICC patients.

Variable	Overall, *n* (%)	Without prior malignancies, *n* (%)	With prior malignancies, *n* (%)	*p* value
Total	9667	8885 (91.91)	782 (8.09)	
Age, years				<0.001
≤65	4546 (47.03)	4317 (48.59)	229 (29.28)	
>65	5121 (52.97)	4568 (51.41)	553 (70.72)	
Sex				<0.001
Male	4793 (49.58)	4332 (48.76)	461 (58.95)	
Female	4874 (50.42)	4553 (51.24)	321 (41.05)	
Race				0.001
White	7663 (79.27)	7014 (78.94)	649 (82.99)	
Black	738 (7.63)	674 (7.59)	64 (8.18)	
Others	1266 (13.10)	1197 (13.47)	69 (8.82)	
Marital status				0.269
Single	3533 (36.55)	3265 (36.75)	268 (34.27)	
Married	5747 (59.45)	5261 (59.21)	486 (62.15)	
Unknown	387 (4.00)	359 (4.04)	28 (3.58)	
AFP				0.001
Negative	2956 (30.58)	2747 (30.92)	209 (26.73)	
Positive	1190 (12.31)	1114 (12.54)	76 (9.72)	
Unknown	5521 (57.11)	5024 (56.54)	497 (63.55)	
AJCC stage				<0.001
I	1697 (17.55)	1481 (16.67)	216 (27.62)	
II	1227 (12.69)	1107 (12.46)	120 (15.35)	
III	2021 (20.91)	1843 (20.74)	178 (22.76)	
IV	2908 (30.08)	2714 (30.55)	194 (24.81)	
Unknown	1814 (18.76)	1740 (19.58)	74 (9.46)	
Tumor size, cm				<0.001
<5 cm	2478 (25.63)	2185 (24.59)	293 (37.47)	
5-8 cm	1920 (19.86)	1792 (30.17)	128 (16.37)	
≥8 cm	2207 (22.83)	2073 (23.33)	134 (17.14)	
Unknown	3062 (31.67)	2835 (31.91)	227 (29.03)	
Vascular invasion				0.006
No	4521 (46.77)	4113 (46.29)	408 (52.17)	
Yes	2252 (23.30)	2084 (23.46)	168 (21.48)	
Unknown	2894 (29.94)	2688 (30.25)	206 (26.34)	
Grade				0.019
I/II	2507 (25.93)	2283 (25.69)	224 (28.64)	
III/IV	1738 (17.98)	1624 (18.28)	114 (14.58)	
Unknown	5422 (56.09)	4978 (56.03)	444 (56.78)	
Treatment				<0.001
None	7075 (73.19)	6574 (73.99)	501 (64.07)	
Local tumor destruction	224 (2.32)	194 (2.18)	30 (3.84)	
Surgery	2285 (23.64)	2041 (22.97)	244 (31.20)	
Unknown	83 (0.86)	76 (0.86)	7 (0.90)	
Radiation				0.293
None	8078 (83.56)	7412 (83.42)	666 (85.17)	
Yes	1537 (15.90)	1423 (16.02)	114 (14.58)	
Unknown	52 (0.54)	50 (0.56)	2 (0.26)	
Chemotherapy				0.019
None/unknown	4054 (41.94)	3695 (41.59)	359 (45.91)	
Yes	5613 (58.06)	5190 (58.41)	423 (54.09)	

Note: ICC: intrahepatic cholangiocarcinoma; AFP: alpha-fetoprotein; AJCC: American Joint Committee on Cancer.

**Table 2:** Univariate and multivariate Cox regression analyses of OS and CSS for ICC patients.

Variable	OS				CSS			
	Univariate analysis		Multivariate analysis		Univariate analysis		Multivariate analysis	
	HR (95% CI)	*p* value	HR (95% CI)	*p* value	HR (95% CI)	*p* value	HR (95% CI)	*p* value
Prior cancers		<0.001		0.002		<0.001		<0.001
No	Reference		Reference		Reference		Reference	
Yes	0.822 (0.752-0.896)		0.870 (0.797-0.950)		0.754 (0.683-0.832)		0.820 (0.741-0.906)	
Age, years		<0.001		<0.001		<0.001		<0.001
≤65	Reference		Reference		Reference		Reference	
>65	1.267 (1.211-1.325)		1.233 (1.177-1.292)		1.267 (1.208-1.329)		1.214 (1.156-1.275)	
Sex		<0.001		<0.001		<0.001		<0.001
Male	Reference		Reference		Reference		Reference	
Female	0.900 (0.860-0.941)		0.891 (0.851-0.933)		0.908 (0.865-0.952)		0.894 (0.852-0.939)	
Race		0.906				0.947		
White	Reference				Reference			
Black	0.986 (0.905-1.075)				0.985 (0.900-1.079)			
Others	0.988 (0.924-1.057)				0.996 (0.928-1.069)			
Marital status		<0.001		0.004		0.001		0.004
Single	Reference		Reference		Reference		Reference	
Married	0.914 (0.872-0.958)		0.950 (0.905-0.997)		0.914 (0.870-0.961)		0.943 (0.896-0.992)	
Unknown	0.875 (0.777-0.986)		0.832 (0.738-0.938)		0.861 (0.757-0.979)		0.826 (0.726-0.940)	
AFP		<0.001		<0.001		<0.001		<0.001
Negative	Reference		Reference		Reference		Reference	
Positive	1.304 (1.212-1.403)		1.166 (1.083-1.255)		1.330 (1.232-1.435)		1.201 (1.112-1.297)	
Unknown	1.159 (1.101-1.219)		1.017 (0.965-1.072)		1.165 (1.104-1.229)		1.030 (1.213-1.402)	
AJCC stage		<0.001		<0.001		<0.001		<0.001
I	Reference		Reference		Reference		Reference	
II	1.433 (1.312-1.566)		1.432 (1.303-1.574)		1.500 (1.364-1.650)		1.486 (1.342-1.645)	
III	1.763 (1.632-1.904)		1.642 (1.510-1.785)		1.849 (1.701-2.010)		1.718 (1.570-1.879)	
IV	2.835 (2.635-3.050)		1.966 (1.812-2.132)		2.968 (2.743-3.211)		2.023 (1.855-2.207)	
Unknown	1.902 (1.758-2.059)		1.348 (1.237-1.469)		1.977 (1.815-2.155)		1.391 (1.267-1.526)	
Tumor size, cm		<0.001		<0.001		<0.001		<0.001
<5 cm	Reference		Reference		Reference		Reference	
5-8 cm	1.235 (1.152-1.325)		1.148 (1.069-1.233)		1.273 (1.181-1.372)		1.169 (1.083-1.261)	
≥8 cm	1.539 (1.439-1.645)		1.223 (1.140-1.311)		1.591 (1.482-1.708)		1.246 (1.157-1.341)	
Unknown	2.066 (1.942-2.197)		1.264 (1.181-1.352)		2.165 (2.027-2.313)		1.304 (1.213-1.402)	
Vascular invasion		<0.001		0.072		<0.001		0.075
No	Reference		Reference		Reference		Reference	
Yes	1.075 (1.015-1.138)		1.000 (0.940-1.064)		1.089 (1.025-1.157)		1.016 (0.953-1.085)	
Unknown	1.318 (1.250-1.390)		0.938 (0.886-0.994)		1.331 (1.259-1.407)		0.942 (0.887-1.001)	
Grade		<0.001		<0.001		<0.001		<0.001
I/II	Reference		Reference		Reference		Reference	
III/IV	1.512 (1.408-1.624)		1.281 (1.193-1.377)		1.523 (1.412-1.642)		1.286 (1.192-1.388)	
Unknown	1.988 (1.879-2.102)		1.178 (1.109-1.253)		2.029 (1.911-2.154)		1.197 (1.123-1.277)	
Treatment		<0.001		<0.001		<0.001		<0.001
None	Reference		Reference		Reference		Reference	
Local tumor destruction	0.426 (0.364-0.499)		0.473 (0.403-0.556)		0.422 (0.355-0.501)		0.469 (0.394-0.559)	
Surgery	0.276 (0.259-0.294)		0.324 (0.301-0.348)		0.263 (0.246-0.282)		0.312 (0.289-0.337)	
Unknown	1.132 (0.905-1.415)		0.995 (0.792-1.250)		1.278 (1.010-1.618)		1.044 (0.821-1.328)	
Radiation		<0.001		<0.001		<0.001		<0.001
None	Reference		Reference		Reference		Reference	
Yes	0.810 (0.761-0.863)		0.816 (0.765-0.869)		0.807 (0.755-0.862)		0.823 (0.770-0.881)	
Unknown	0.854 (0.620-1.174)		1.078 (0.783-1.484)		0.847 (0.608-1.181)		1.111 (0.796-1.550)	
Chemotherapy		0.037		<0.001		0.023		<0.001
None/unknown	Reference		Reference		Reference		Reference	
Yes	0.952 (0.910-0.997)		0.694 (0.660-0.730)		0.945 (0.900-0.992)		0.671 (0.636-0.708)	

Note: ICC: intrahepatic cholangiocarcinoma; AFP: alpha-fetoprotein; AJCC: American Joint Committee on Cancer; HR: hazard ratio; CI: confidence interval; OS: overall survival; CSS: cancer specific survival.

**Table 3:** Multivariate Cox regression analysis of OS and CSS in ICC patients stratified by initial prior cancer site.

Characteristics	OS		CSS	
	HR (95% CI)	*p*	HR (95% CI)	*p*
Prior cancer site (vs. <none)				
Breast	0.797 (0.630-1.008)	0.059	0.748 (0.574-0.975)	0.032
Colon and rectum	0.886 (0.791-1.120)	0.312	0.856 (0.640-1.145)	0.294
Prostate	0.865 (0.729-1.027)	0.098	0.786 (0.649-0.953)	0.014
Bladder	1.298 (0.949-1.774)	0.102	1.204 (0.849-1.708)	0.297
Liver/gallbladder/other bile duct	0.870 (0.611-1.240)	0.442	0.829 (0.567-1.210)	0.331
Oral cavity and pharynx	0.809 (0.537-1.219)	0.311	0.737 (0.469-1.157)	0.185
Melanoma	1.021 (0.732-1.424)	0.903	0.900 (0.621-1.306)	0.581
Uteri	0.864 (0.583-1.281)	0.468	0.903 (0.568-1.437)	0.668
Non-Hodgkin lymphoma	0.728 (0.491-1.080)	0.115	0.737 (0.469-1.157)	0.185
Kidney	1.438 (0.936-2.209)	0.097	1.476 (0.889-2.452)	0.133

Note: ICC: intrahepatic cholangiocarcinoma; HR: hazard ratio; CI: confidence interval; OS: overall survival; CSS: cancer specific survival.

## Data Availability

All the data in the current study are publicly available in the Surveillance, Epidemiology, and End Results database (https://seer.cancer.gov/).

## Supplementary Materials

Supplementary 1 Figure S1: distribution of causes of death for ICC patients with a history of prior malignancy. ICC: intrahepatic cholangiocarcinoma; ICC-PM: intrahepatic cholangiocarcinoma with prior malignancy; OICC: patients only with intrahepatic cholangiocarcinoma.

Supplementary 2 Figure S2: OS of subgroups stratified by AJCC stage. (A) AJCC stage I, (B) AJCC stage II, (C) AJCC stage III, and (D) AJCC stage IV. OS: overall survival; AJCC: American Joint Committee on Cancer.

Supplementary 3 Figure S3: the CSS of subgroups stratified by AJCC stage. (A) AJCC stage I, (B) AJCC stage II, (C) AJCC stage III, and (D) AJCC stage IV. OS: overall survival; AJCC: American Joint Committee on Cancer.

Supplementary 4 Table S1: the 1-, 3-, and 5-year OS and CCS in ICC patients with and without prior cancers.

## References

[1] Mukkamalla S. K. R., Naseri H. M., Kim B. M., Katz S. C., Armenio V. A. (2018). Trends in incidence and factors affecting survival of patients with cholangiocarcinoma in the United States. *Journal of the National Comprehensive Cancer Network*.

[2] Saha S. K., Zhu A. X., Fuchs C. S., Brooks G. A. (2016). Forty-year trends in cholangiocarcinoma incidence in the U.S.: intrahepatic disease on the rise. *The Oncologist*.

[3] Mavros M. N., Economopoulos K. P., Alexiou V. G., Pawlik T. M. (2014). Treatment and prognosis for patients with intrahepatic cholangiocarcinoma: systematic review and meta-analysis. *JAMA Surgery*.

[4] Bluethmann S. M., Mariotto A. B., Rowland J. H. (2016). Anticipating the "silver tsunami": prevalence trajectories and comorbidity burden among older cancer survivors in the United States. *Cancer Epidemiology, Biomarkers & Prevention*.

[5] Keegan T. H. M., Bleyer A., Rosenberg A. S., Li Q., Goldfarb M. (2017). Second primary malignant neoplasms and survival in adolescent and young adult cancer survivors. *JAMA Oncology*.

[6] Hayat M. J., Howlader N., Reichman M. E., Edwards B. K. (2007). Cancer statistics, trends, and multiple primary cancer analyses from the Surveillance, Epidemiology, and End Results (SEER) program. *The Oncologist*.

[7] Murphy C. C., Gerber D. E., Pruitt S. L. (2018). Prevalence of prior cancer among persons newly diagnosed with cancer: an initial report from the Surveillance, Epidemiology, and End Results program. *JAMA Oncology*.

[8] Haanen J., Ernstoff M., Wang Y. (2020). Rechallenge patients with immune checkpoint inhibitors following severe immune-related adverse events: review of the literature and suggested prophylactic strategy. *Journal for Immunotherapy of Cancer*.

[9] Mahvi D. A., Liu R., Grinstaff M. W., Colson Y. L., Raut C. P. (2018). Local cancer recurrence: the realities, challenges, and opportunities for new therapies. *CA: a Cancer Journal for Clinicians*.

[10] Joung J. Y., Lim J., Oh C. M. (2015). Risk of second primary cancer among prostate cancer patients in Korea: a population-based cohort study. *PLoS One*.

[11] Yang Y., Yang Y., Yan S. (2021). Risk and survival of second primary malignancies following diagnosis of gastric mucosa-associated lymphoid tissue lymphomas: a population-based study. *Current Problems in Cancer*.

[12] Laccetti A. L., Pruitt S. L., Xuan L., Halm E. A., Gerber D. E. (2015). Effect of prior cancer on outcomes in advanced lung cancer: implications for clinical trial eligibility and accrual. *Journal of the National Cancer Institute*.

[13] Gerber D. E., Laccetti A. L., Xuan L., Halm E. A., Pruitt S. L. (2014). Impact of prior cancer on eligibility for lung cancer clinical trials. *Journal of the National Cancer Institute*.

[14] Bian X., He X., Yang L., Wu W., Li L. (2020). Prognosis of hepatocellular carcinoma among cancer survivors with other types of primary tumors. *Digestive Diseases and Sciences*.

[15] He X., Li Y., Su T. (2018). The impact of a history of cancer on pancreatic ductal adenocarcinoma survival. *United European Gastroenterology Journal*.

[16] NCI Cancer Treatment Evaluation Program (2021). *guidelines regarding the inclusion of cancer survivors and HIV-positive individuals on clinical trials*.

[17] Bian X., Wang K., Wang Q. (2021). The impact of a prior malignancy on outcomes in gastric cancer patients. *Cancer Medicine*.

[18] Sung H., Ferlay J., Siegel R. L. (2021). Global cancer statistics 2020: GLOBOCAN estimates of incidence and mortality worldwide for 36 cancers in 185 countries. *CA: a Cancer Journal for Clinicians*.

[19] Loibl S., Poortmans P., Morrow M., Denkert C., Curigliano G. (2021). Breast cancer. *Lancet*.

[20] Dekker E., Tanis P. J., Vleugels J. L. A., Kasi P. M., Wallace M. B. (2019). Colorectal cancer. *Lancet*.

[21] Attard G., Parker C., Eeles R. A. (2016). Prostate cancer. *Lancet*.

[22] Miller K. D., Nogueira L., Mariotto A. B. (2019). Cancer treatment and survivorship statistics, 2019. *CA: a Cancer Journal for Clinicians*.

[23] Zhou Y., Guan H., Fu Y. (2018). The impact of pre-existing cancer on survival of prostate cancer patients: a population-based study. *Medicine*.

[24] Jia H., Li Q., Yuan J., Sun X., Wu Z. (2020). Second primary malignancies in patients with colorectal cancer: a population-based analysis. *The Oncologist*.

[25] Yang J., Wei R., Song X. (2021). Risk of second primary malignancy after minor salivary gland cancer: a Surveillance, Epidemiology, and End Results database analysis. *Head & Neck*.

[26] Xu X. F., Xing H., Han J. (2019). Risk factors, patterns, and outcomes of late recurrence after liver resection for hepatocellular carcinoma: a multicenter study from China. *JAMA Surgery*.

[27] Ladigan-Badura S., Vangala D. B., Engel C. (2021). Value of upper gastrointestinal endoscopy for gastric cancer surveillance in patients with Lynch syndrome. *International Journal of Cancer*.

[28] Pruitt S. L., Laccetti A. L., Xuan L., Halm E. A., Gerber D. E. (2017). Revisiting a longstanding clinical trial exclusion criterion: impact of prior cancer in early-stage lung cancer. *British Journal of Cancer*.

[29] Pan D., Xu W., Gao X., Yiyang F., Wei S., Zhu G. (2021). Survival outcomes in esophageal cancer patients with a prior cancer. *Medicine*.

[30] Kim E. S., Bruinooge S. S., Roberts S. (2017). Broadening eligibility criteria to make clinical trials more representative: American Society of Clinical Oncology and Friends of Cancer Research Joint Research Statement. *Journal of Clinical Oncology*.

[31] Patel T. (2001). Increasing incidence and mortality of primary intrahepatic cholangiocarcinoma in the United States. *Hepatology*.

[32] Sripa B., Kaewkes S., Sithithaworn P. (2007). Liver fluke induces cholangiocarcinoma. *PLoS Medicine*.

