# Supplementary figures and images for: The Impact of a History of Different Other Cancers on the Long-Term Outcomes of Patients with Intrahepatic Cholangiocarcinoma: A Population-Based Analysis
Source: Biomed Res Int. 2022 Feb 25;2022:3970884. doi: 10.1155/2022/3970884 (PMC8897745; doi:10.1155/2022/3970884)

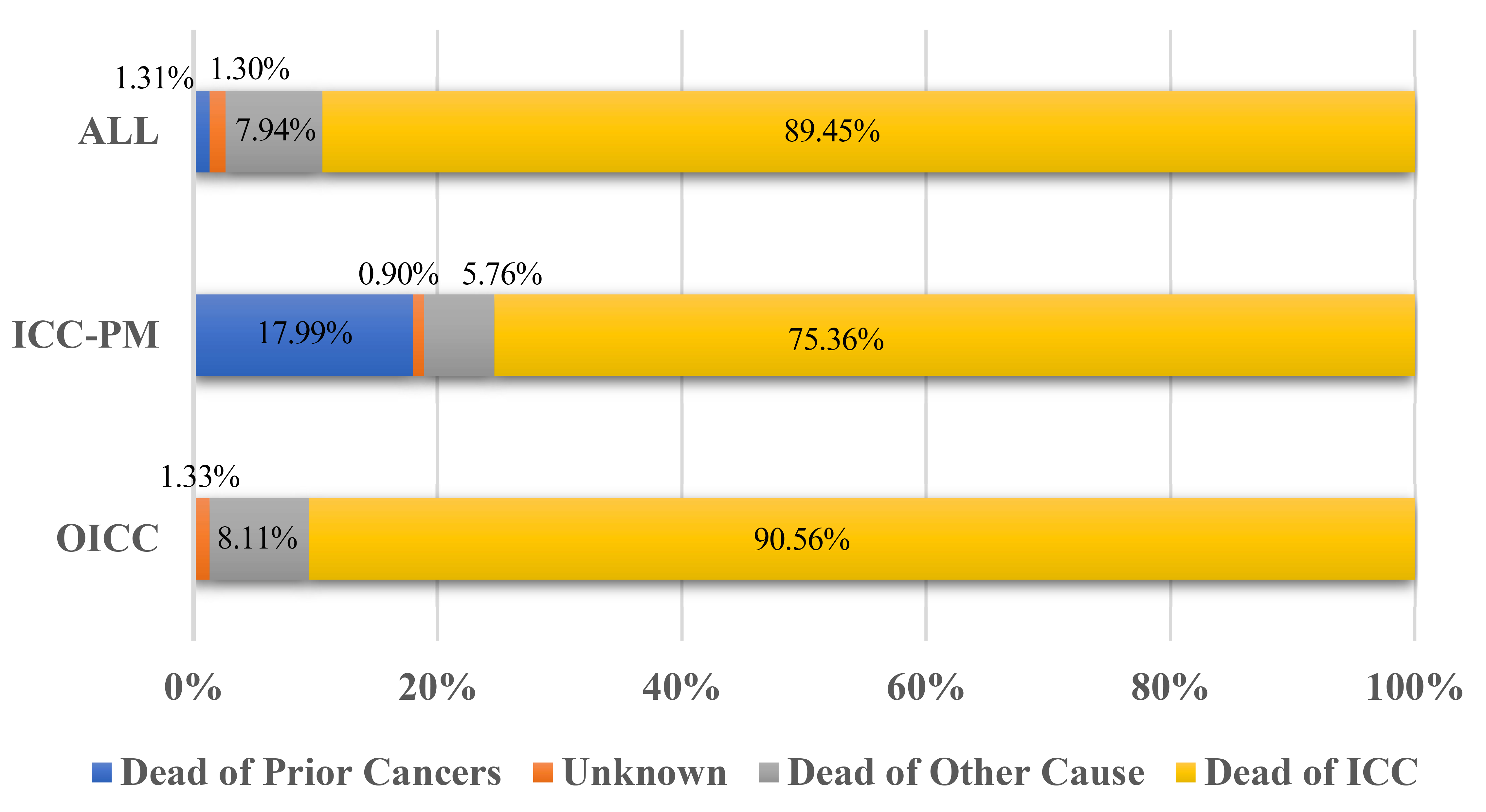

Supplement: Supplementary 1 — Figure S1: distribution of causes of death for ICC patients with a history of prior malignancy. ICC: intrahepatic cholangiocarcinoma; ICC-PM: intrahepatic cholangiocarcinoma with prior malignancy; OICC: patients only with intrahepatic cholangiocarcinoma. [file 3970884.f1.png]

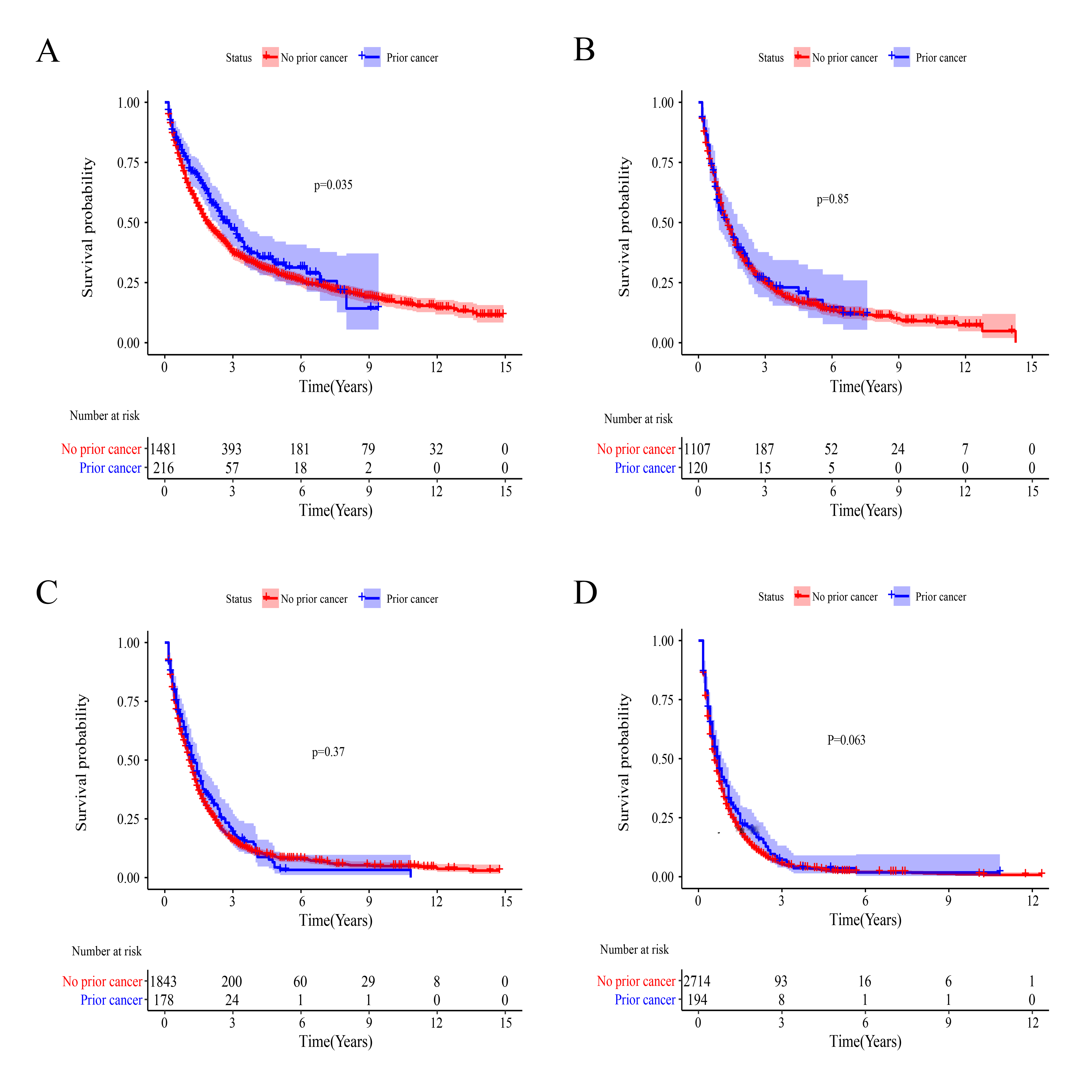

Supplement: Supplementary 2 — Figure S2: OS of subgroups stratified by AJCC stage. (A) AJCC stage I, (B) AJCC stage II, (C) AJCC stage III, and (D) AJCC stage IV. OS: overall survival; AJCC: American Joint Committee on Cancer. [file 3970884.f2.png]

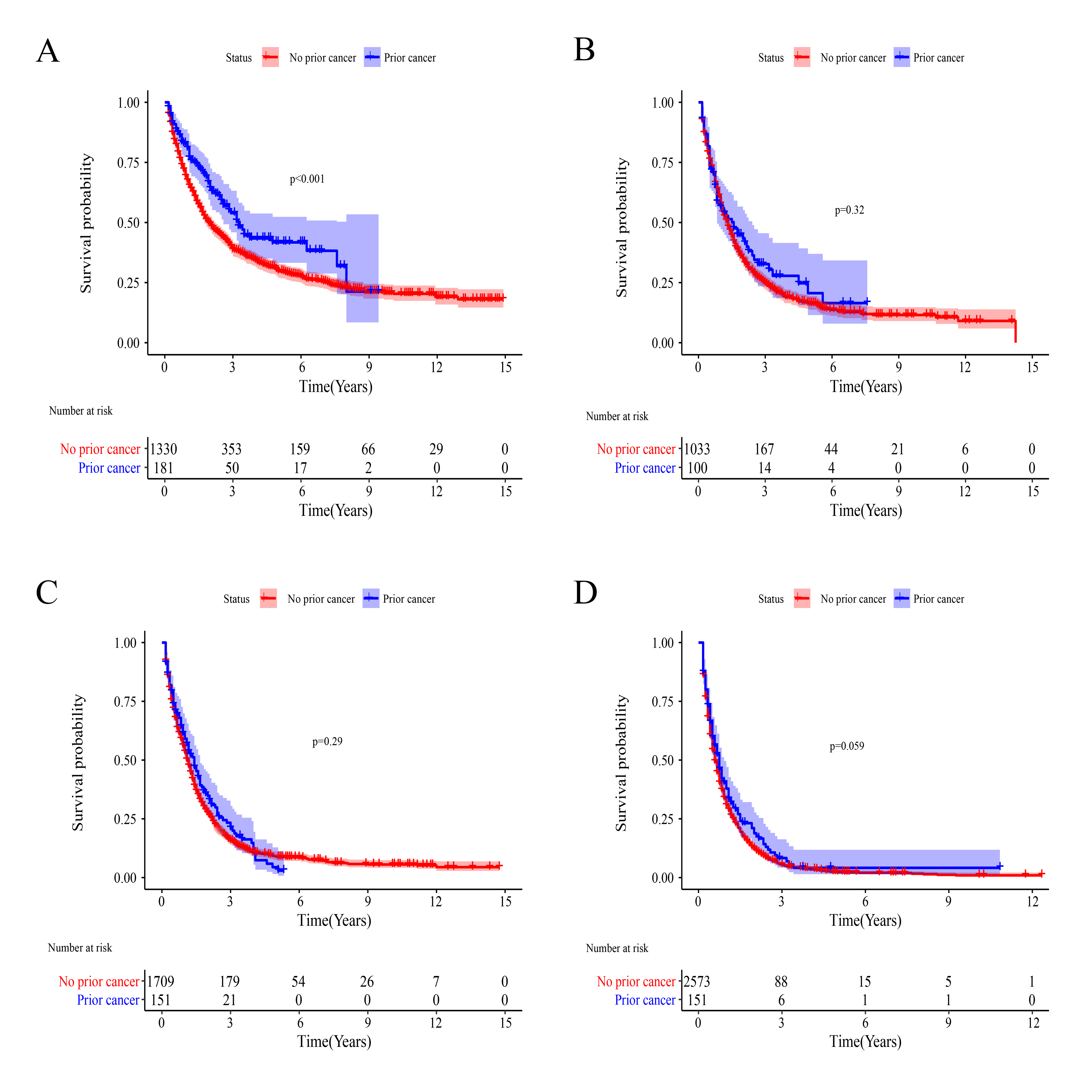

Supplement: Supplementary 3 — Figure S3: the CSS of subgroups stratified by AJCC stage. (A) AJCC stage I, (B) AJCC stage II, (C) AJCC stage III, and (D) AJCC stage IV. OS: overall survival; AJCC: American Joint Committee on Cancer. [file 3970884.f3.png]
