# Supplementary material for: The Impact of a History of Different Other Cancers on the Long-Term Outcomes of Patients with Intrahepatic Cholangiocarcinoma: A Population-Based Analysis
Source: Biomed Res Int. 2022 Feb 25;2022:3970884. doi: 10.1155/2022/3970884 (PMC8897745; doi:10.1155/2022/3970884)
Supplement: Supplementary 4 — Table S1: the 1-, 3-, and 5-year OS and CCS in ICC patients with and without prior cancers. [file 3970884.f4.docx]

Table S1 The 1-, 3- and 5-year OS and CCS in ICC patients with and without prior cancers

| Year | OS | CSS |
| --- | --- | --- |
| 1- |  |  |
| With prior cancers | 56.2 % (52.8%-60.0%) | 58.8% (55.0%-62.9%) |
| Without prior cancers | 46.6% (45.6%-47.7%) | 47.1% (46.0%-48.2%) |
| 3- |  |  |
| With prior cancers | 24.7% (21.4%-28.5%) | 29.1% (25.2%-33.6%) |
| Without prior cancers | 18.1% (17.2%-19.0%) | 18.3% (17.4%-19.3%) |
| 5- |  |  |
| With prior cancers | 14.0% (11.1%-17.7%) | 17.4% (13.8%-22.1%) |
| Without prior cancers | 11.8% (11.0%-12.7%) | 12.2% (11.4%-13.1%) |

Note: ICC, intrahepatic cholangiocarcinoma; OS, overall survival; CSS, cancer specific survival.
